# The SEEKING Drive and Its Fixation: A Neuro-Psycho-Evolutionary Approach to the Pathology of Addiction

**DOI:** 10.3389/fnhum.2021.635932

**Published:** 2021-08-12

**Authors:** Antonio Alcaro, Anthony Brennan, David Conversi

**Affiliations:** ^1^Department of Psychology, Sapienza University of Rome, Rome, Italy; ^2^Independent Researcher, Zurich, Switzerland

**Keywords:** SEEKING, dopamine, mesolimbic, addiction, compulsive, habit, exploration, drive

## Abstract

Neuro-ethological studies conducted by Panksepp and his colleagues have provided an understanding of how the activity of the mesolimbic dopaminergic (ML DA) system leads to the emotional disposition to SEEK/Explore, which is involved in all appetitive motivated behavior and mental activity. In pathological addiction phenomena, this emotional disposition “fixes” itself on certain obsessive-compulsive habits, losing its versatility and its natural predisposition to spontaneous and unconditioned activation. Overall, the result is a consistent disinterest in everything that is not the object of addiction. From a neuro-psycho-evolutionary point of view, the predisposition to develop addictive behavior can be attributed to a loss of “functional autonomy” of the SEEKING/Explorative disposition. Indeed, as shown by animal and human studies, the tendency to be conditioned by situations and contexts that provide an immediate reward can be closely related to a deficit in the tonic endogenous activity of the ML DA-SEEKING system.

## Introduction

According to the American Society of Medicine (American Society of Addiction Medicine, 2011), “Addiction is a treatable, chronic medical disease involving complex interactions among brain circuits, genetics, the environment, and an individual’s life experiences. People with addiction use substances or engage in behaviors that become compulsive and often continue despite harmful consequences.” The literature on this phenomenon is immense and reviewing that comprehensively is beyond the scope of this article, which is to conceptually analyze the potential contribution to the understanding of addiction of the neuro-psycho-evolutionary approach to the SEEKING drive and its fixation.

From a neuro-psycho-evolutionary perspective, SEEKING is a neurobiologically based and evolutionarily conserved emotional drive (Ikemoto and Panksepp, 1999; Alcaro et al., 2007; Alcaro and Panksepp, 2011). Such drive is thought to be involved in all the appetitively motivated behaviors and play an essential role in stimulating mental activity. Addiction can be conceptualized as the result of SEEKING becoming “fixated” upon compulsive habits, thereby losing its versatility and spontaneous activation to novelty, regardless of the presence of other reinforcements. This fixation results in a lack of interest in everything other than the target of addiction. Environments that provide plentiful and accessible conditioned rewards, while not stimulating unconditioned exploration toward novel and unpredictable settings, further exacerbated the loss of “functional autonomy” of the SEEKING drive.

Substance-related addictions, such as those to alcohol or drugs, almost always have clear and recognizable signs. Usually, the addicted tend to isolate themselves or restrict their acquaintances to a small circle of people with whom they share the same drug-related habits (McAlaney et al., 2021). Such social maladjustment often leads to marginalization and antisocial behavior (Fox et al., 2013). In contrast, non-substance-related forms of addiction, such as those to the Internet, food, gambling, sex, work, etc., are often more challenging to identify because they arise as common behaviors within everyday social contexts. They are more subtle and devious, and it can be complicated to determine whether you are dealing with addictive behavior (Alavi et al., 2012).

Consider, as a paradigmatic example, the following clinical sketch. A few weeks ago, a woman decided for the first time to consult a psychotherapist about a problem she was having with her 17-year-old daughter. The daughter had been behaving atypically for several months: she would lock herself in her room for hours, stay awake at night, and catch up on lost sleep after returning from school in the afternoon. Questioned by her mother, the girl claimed to spend all time in the room, in front of the computer or on her mobile phone, “chatting” with friends or browsing various social networks. Are we facing the symptoms of true Internet addiction, or can we consider the girl’s smartphone overuse in line with her lifespan and socio-cultural context without overemphasizing it?

Although dependent habits are widespread, we need to develop a better distinction between behaviors that resemble addiction but do not compromise the individual’s overall functioning and psychophysical health, and maladaptive, compulsive rituals, which require intervention. To this aim, it may be helpful to view addiction from an evolutionary perspective, using the contributions from comparative research on animal brains and behavior. Such studies have identified the mesolimbic DAergic system as the neural substrate fundamental to establishing and maintaining all forms of addiction (for review Kuhn et al., 2019). Over the years, a more extensive corticolimbic circuit model has been developed that now includes other brain regions involved in compulsion and decision-making processes (e.g., the orbitofrontal cortex). This extended model can better capture the often-stochastic transition toward compulsive use, integrating the genetic and epigenetic information underlying vulnerability to addiction (Volkow et al., 2019; Lüscher and Janak, 2021). However, the dopamine-mesolimbic motivation-reward-reinforcement cycle remains the most coherent physiological theory in addiction (Popescu et al., 2021). Abundant animal and human studies have shown that the predisposition to develop addictive behaviors can be closely related to a deficit in the endogenous tonic activity of the ML DA system (George et al., 1995; Marinelli and White, 2000; Chefer et al., 2003; Alcaro et al., 2007).

Interestingly, although the neurobiological processes responsible for the development and maintenance of dependent behaviors are common to both humans and many animal species, the phenomenon of pathological addiction only manifests itself under certain conditions where predisposing factors result in vulnerability, suggesting an interaction between biological and socio-cultural factors (Westermeyer, 1999).

Based on this premise, we propose a hypothesis that attempts to identify a psycho-biological substrate for individual vulnerability to the development of pathological addiction and connect such predisposition to specific environmental, cultural, and socio-economic factors that characterize our contemporary world.

## The ML DA-SEEKING System

Studies of the neurobiological processes involved in the establishment of addictive behavior converge with the dominant role played by the ML DA system and its associated areas and circuits (Wise and Bozarth, 1987; Di Chiara and Imperato, 1988; White, 1996, but see Alcaro et al., 2007 for a summary on the subject). The activation of this system is indeed responsible for the behaviorally incentivizing effects of rewarding stimuli and influences learning, conditioning the incentive value attributed to the stimuli and behaviors associated with the process of reinforcement (classical conditioning and operant conditioning) (Robinson and Berridge, 2001, 2003, 2008; Salamone and Correa, 2002; Everitt and Robbins, 2005). Traditionally referred to as the “reward circuit” (Wise, 2002), the ML DA system, therefore, plays a fundamental role in acquiring compulsive habits and in the frequent reactivation of these habits by conditioned stimuli.

To explain this phenomenon, Robinson and Berridge proposed the incentive-sensitization theory more than 20 years ago (1993). According to this theory, repeated exposure to artificial reinforcers, such as drugs of abuse, sensitize the ML DA system to these reinforcers and to the associated conditioned stimuli, thereby enhancing their motivational value (Vanderschuren and Pierce, 2010). Subsequently, the frequent repetition of behaviors that have produced these reinforcements gradually shapes cognitive and motor procedural schemata, which in turn become actual compulsive habits (Robbins and Everitt, 1999).

Following a highly simplified sketch, we can hypothesize that the ML DA system acts as a link between the representation of specific stimuli and environmental contexts, associated with reinforcements by some limbic structures, such as the amygdala or hippocampus, and a series of procedural sequences (cognitive and motor) processed within the frontal cortico-subcortical circuits (see Alcaro et al., 2007 for a summary).

From a neuro-ethological perspective dealing with the unconditional factors that influence learning (Lorenz, 1965; Panksepp, 1998), we can understand how the ML DA system is the neurobiological substrate of a primary emotional drive known as the SEEKING/Exploration disposition. It is an intrinsic psycho-behavioral function of the brain that has evolved to make animals explore and seek all kinds of stimuli necessary for survival and reproduction (Ikemoto and Panksepp, 1999; Alcaro et al., 2007; Alcaro and Panksepp, 2011). To be precise, SEEKING is the instinctive basis for all motivated behavior in the appetitive phase (e.g., the search for distal stimuli) which anticipates the second phase of consumption (regarding proximal stimuli) (Wise and Bozarth, 1987; Berridge and Robinson, 1998; Salamone and Correa, 2012).

The SEEKING drive perspective connects with another enormous research fields: the one about intrinsic vs. extrinsic motivation (e.g., Santucci et al., 2019), which – in turn – relates to the information-SEEKING research topic (e.g., Horan et al., 2019). Although there are many interpretations about the roles of the ML DA system (e.g., Bromberg-Martin et al., 2010; FitzGerald et al., 2015; Schultz, 2015), the SEEKING drive theory has the advantage of subsuming all other functions within a single basic psycho-behavioral disposition (see Alcaro et al., 2007 for a review). From a behavioral perspective, the SEEKING disposition expresses itself as locomotion, sensory exploration, orientation reflexes, and approach movements. From a subjective point of view, however, this disposition is accompanied by feelings of enthusiasm, desire, interest, curiosity, and trust, as well as mental states of expectation that anticipate the existence of future rewards (Alcaro and Panksepp, 2011; Cruciani et al., 2011; Conversi et al., 2014).

From a neuro-psychoanalytical perspective, the ML DA system has been identified with the cerebral substrate of libido, considered not only as sexual energy but also as a source of outwardly directed energy investment (Kaplan-Solms and Solms, 2000; see Appendix A).

Remarkably, invertebrates also have a SEEKING/Exploration disposition, which is regulated by neurochemical processes like those of mammals. For example, the administration of cocaine and amphetamines, drugs that act directly on DA transmission, can influence the behavior of freshwater crayfish in very similar ways to that of mammals (Alcaro et al., 2011). Crayfishes are invertebrates that, in a familiar environment and without specific stimulation, spend most of their time in a state of complete inactivity. However, as soon as psychostimulants are administered into their circulatory system, they activate and begin exploring the surroundings as if it has suddenly become new and interesting. Activating effects even stronger arise by administering the psychostimulants directly into the ganglion of the head, the brain equivalent in vertebrates (Alcaro et al., 2011).

The results suggest that these animals are susceptible to the incentive effect of these drugs of abuse. Moreover, they prefer drug-associated environments and learn to act to obtain their administration (Søvik and Barron, 2013; Zhu et al., 2014; Engleman et al., 2016; Kaun and Rothenfluh, 2017; Huber et al., 2018). Thus, the addiction phenomenon seems to be rooted in psycho-neuro-behavioral functions that are very ancient from a phylogenetic perspective and are indeed related to the SEEKING/Exploration emotional disposition.

## Directed vs. Undirected SEEKING

The SEEKING emotional disposition typically strives toward specific objects. When recruited by basic biological impulses, such as hunger, thirst, sexuality, the need to find shelter, etc., it promotes exploration to achieve a specific purpose, which coincides with a particular goal-object or environmental condition capable of satisfying the urge. SEEKING ceases when the organic conditions that led to the recruitment of the drive end.

While recruited by basic biological drives and conditioned by homeostatic-visceral regulatory mechanisms, the SEEKING/Exploration system retains some functional autonomy. Indeed, the electrical stimulation of the SEEKING/Exploration circuits activate motivated behaviors related to eating, drinking, or hunting, depending on the environmental contexts in which it is administered (Valenstein et al., 1969). Moreover, under conditions where reinforcing cues or stimuli are absent, the electrical stimulation produces generalized excitation and unspecific exploratory behaviors (Gallistel, 1974; Panksepp, 1998). Thus, unspecific exploration and appetitive motivation may be the purest expressions of SEEKING disposition (Ikemoto and Panksepp, 1999; Alcaro et al., 2007; Alcaro and Panksepp, 2011).

However, through learning, the autonomous expression of SEEKING can be narrowed down, restricting the boundaries of its operation, and directing exploration to specific objects and particular procedures. In this case, the activation of the SEEKING emotional disposition is channeled to a specific sequence of activities aimed at achieving a particular stimulus reinforcement (food) and stops when the environmental conditions that led to its activation are no longer present. For example, a rat that has learned to walk through a maze to obtain food will execute a particular sequence of movements along the tunnels of the experimental apparatus until reaching the goal. The learning process, both in terms of stimulus (classical conditioning) and behavior (operant conditioning), determines a gradual and progressive removal of the functionally autonomous component of the SEEKING/Exploration system, linked to the ventromedial cortico-striatal areas of the DA system and a greater recruitment of the dorsal-lateral regions of the same circuits (Everitt and Robbins, 2005; Alcaro et al., 2007). Through reiteration, this process leads to an increasingly automated execution of SEEKING-driven behavioral and mental activities, structuring them into complexes of habits that can occur with low consciousness and intentionality. In the next section, we will investigate the extent to which such habits could play a decisive role in addiction.

## From Conditioning to Habits

The initial experimental research on addiction explained it as a neurochemical imbalance due to repeated drug use motivated to avoid withdrawal symptoms (Solomon, 1977). Over time, however, it has become increasingly clear that, while withdrawal symptoms disappear in a few weeks, addicts remain prone to “craving,” i.e., a hard-to-repress urge that drives them to act compulsive behaviors, triggered by drug-associated cues even after a long abstinence. Since then, research has increasingly focused on craving-induced relapse and its underlying mechanisms (Shaham et al., 2003; Hyman, 2005).

In the last thirty years, the primary focus of theoretical and experimental investigation has become the phenomenon of craving, which is explained by two interrelated processes, one general and one specific. The general process comprises deficits in impulses’ inhibition and cognitive control, i.e., reflectively weighing up the consequences of one’s actions (Volkow and Fowler, 2000; Jentsch et al., 2014). The specific process involves the conditioning through which mental and behavioral processes come under the control of certain kinds of memories and habits that relate to the contexts of addiction-associated stimuli and behaviors (White, 1996; Everitt and Robbins, 2005; Hyman et al., 2006). For example, such memories could relate to bars or gambling halls for a slot machine player, certain sounds and images associated with slot machines, and the gestures performed while playing.

In this paragraph, we will specifically address the second of these processes: the specific one, postponing the discussion of the general one to the next section. Although distinct, the two processes are deeply interrelated: it is easier to succumb to cue-induced craving when the capacity for reflective self-regulation is diminished, while being subjected to powerful conditioned associations can, over time, weaken the ability for reflective self-regulation.

Neurobiological research based on animal models of drug addiction has shown that the intake of abused substances leads to a gradual functional reorganization of the brain from the molecular to the systemic-circuit level (Nestler, 2002, 2014). The neurobiological and psycho-behavioral processes identified by these studies turn out to be broadly similar in other forms of addiction that do not involve drugs. What has emerged from this research is that addiction implies a process of maladaptive learning, characterized by the progressive acquisition of compulsive habits, which plunge the individual into a cyclical spiral of anguish and despair (Koob and Le Moal, 1997, 2001, 2008). Forming a constricting nexus, the set of memories and compulsive habits end up dominating, increasingly exclusively, the needs, motivations, and desires of the addict. Parallelly, all other activities and contexts lose their ability to stimulate even the minimum interest.

From an Affective Neuroscience viewpoint (Panksepp, 1998; Alcaro and Panksepp, 2011), addictions can be characterized by narrowing and tightening of the SEEKING disposition, which is activated only in certain contexts and channeled through specific sequences of procedural activities. All this can be orchestrated by a form of top-down control exerted by superior cortical and limbic structures on the subcortical centers’ activity, especially the ML DA system (Alcaro et al., 2007). Such top-down control may reflect increased glutamatergic transmission in nodal centers of cortical-striatal circuits (Pennartz et al., 2009), underlying the sensitization process by which addiction memories gain disproportionate incentive power over behavior (Steketee and Kalivas, 2011). In turn, increased descending excitatory transmission may suppress the endogenous activity of SEEKING/Exploration systems, thus compromising its functional autonomy. The loss of functional autonomy of the SEEKING system may constitute the common root of all forms of addiction. Nevertheless, different addictive behavior depends on the action of distinct memory complexes.

## Individual Vulnerability as an Endogenous Deficit of the ML DA-SEEKING System

There is widespread agreement that vulnerability to addiction involves epigenetic regulation of so-called endophenotypes (Nestler, 2013; Walker and Nestler, 2018). Originating from the research conducted by Panksepp and his collaborators, it has been possible to characterize these endophenotypes based on the organization and expression of the basic emotional systems (Panksepp, 2006). Among the addiction-related endophenotypes, here, we focus specifically on an endogenous deficit in the ML DA-SEEKING system, although we do not exclude the intervention of anomalies in other emotional systems.

The loss of functional autonomy of the ML DA-SEEKING system is almost certainly accelerated and reinforced by the conditioning experiences that gradually lead to addiction. However, it is plausible that the system is already vulnerable to addiction even before such events. Such a hypothesis would explain why some people are fascinated by situations and environments where it is easy to develop an addiction, while others stay away from or are not attracted to them. From this point of view, the development of a specific addiction may reflect an exacerbation of existing tendencies and individual susceptibilities to conditioning.

Indeed, animal models of addiction have shown that the tonic hypofunctionality (the lowered tonic background activity) of the ML DA system is a crucial factor in both the predisposition to addiction and the maintenance of compulsive behaviors. From a neurochemical point of view, tonic DA transmission extends outside the synaptic space and changes slowly, because it is relatively independent of nerve impulses (Grace, 2000). As a result of inhibitory regulatory processes mediated by DA autoreceptors, DA’s tonic levels inhibit the readiness for the electrical discharge of DA neurons (Grace, 2000; Schmitz et al., 2003). Tonic DA levels also appear to promote an increase in phasic DA release from each single firing pulse (Alcaro et al., 2007). Therefore, the tonic levels of DA express the ratio between discharge potency (the number of DA molecules released by each impulse) and electric excitability of DA neural cells. Tonic DA concentrations represent the degree of functional autonomy of the ML DA system and its independence from excitatory (glutamatergic) stimulation. Coherently, animal models have confirmed that subjects vulnerable to drug addiction exhibit hypoactivity in tonic DA transmission and hyperactivity of ML DA neurons (George et al., 1995; Marinelli and White, 2000; Chefer et al., 2003; Alcaro et al., 2007).

From a psychological point of view, losing functional autonomy of the SEEKING/Exploration system is linked to two closely connected phenomena:

(1) A predisposition toward an anhedonic depressive mood characterized by a lack of enthusiasm and a tendency toward distrust and renunciation (Gold et al., 2018; Salone, 2018; Szczypiński and Gola, 2018).
(2) An emotional and behavioral hyper-reactivity to environmental conditions characterized by novelty or artificial reinforcements such as drugs of abuse (Piazza et al., 1989; Pierre and Vezina, 1997). Individuals characterized by this hyper-reactivity have been classified as “novelty seekers” or “sensation seekers” (Bardo et al., 1996).

According to the self-medication hypothesis, such individuals are prone to search for novelty and develop addictive behaviors to provide accessible, rapid, and powerful stimulation that compensates for an endogenous motivational defect (Markou et al., 1998). Unfortunately, this stimulation offers temporary relief but ends up fueling depressive withdrawal, as the potent and circumscribed stimulation of the SEEKING/Exploration system fuels an inhibition of its tonic dopaminergic activity.

## Predisposing Environmental Factors

Research conducted on animals and humans has revealed that an individual predisposition to addiction is promoted by stressful environmental conditions (see Ruisoto and Contador, 2019 for a summary). It has also been shown in animals that this predisposition is markedly attenuated if the animals are raised in an enriched environment, characterized by exposure to new stimuli, social interactions, and physical exercise (see Crofton et al., 2015 for a summary of research on the topic). Amongst the various stressors, those related to adverse social conditions or events have received particular attention, as they are probably the most relevant to human psychological development (Pelloux et al., 2019). Social isolation, deprivation from parental care, and forced submission are the elements of social stress that most predispose to the development of addiction.

From a psychobiological perspective, we can define stress as environmental influences that exceed the individual’s coping capacity and exert pressure on him to force certain activities and restrict his freedom of action. It is a constraint on the spontaneous dispositions that animate from within, forcing the individual to adapt to the environment, limiting the self’s free and spontaneous expression (Winnicott, 1965). The growth and development of a healthy and genuine personality are directly linked to the expression of positive emotional dispositions, such as SEEKING/Exploration, PLAY, LUST/Sexuality, and CARE. The expression of these dispositions can be generally inhibited by living conditions, stressful or traumatic events, or conditions that involve emotional neglect.

Neurochemical evidence shows that traumatic or stressful events activate the glutamatergic circuits at the cortical and limbic levels. Beyond a certain threshold, this activation triggers inflammatory and cytotoxic processes that alter the functionality of the neuronal connectivity (Averill et al., 2017). Repeated, chronic, and uncontrollable stresses also cause a hypofunctionality of the ML DA system. Such hypofunctionality has been linked to a motivational deficit manifested as a reluctance to face and resolve negative situations (learned helplessness) and diminished interest in pleasing situations (anhedonia) (Cabib and Puglisi-Allegra, 1996; Gold et al., 2018; Stanton et al., 2019).

In summary, stressful environmental conditions, especially social ones, may predispose vulnerable individuals to develop an addiction by inhibiting the spontaneous expression of positive emotional dispositions (SEEKING, PLAY, CARE, and LUST). Indeed, those emotions promote and reinforce the influence that the individual exerts on his environment. On the other hand, the pressure that the environment exerts on the individual restricts the range of its actions and favors establishing compulsive habits that act autonomously and independently of the expression of subjective intentionality.

## A Neuro-Psycho-Evolutionary Interpretation of the Phenomena of Addiction

The evolution of terrestrial vertebrates is characterized by an increasing degree of encephalization and by the development of an extensive and densely connected cortical mantle. The process of corticalization begins to be visible in reptiles, which have an allocortical formation known as the medial cortex. This formation is considered the anatomical precursor of the medial temporal lobe of mammals. The medial temporal lobe, which also includes the hippocampus and the parahippocampal gyrus, is the oldest part of the mammalian cortex in evolutionary terms and is involved in the processes of spatial navigation and the formation of abstract representations necessary for spatio-temporal and declarative memory, contextual learning, and some forms of social cognition (Reiter et al., 2017). The evolution of the cerebral cortex has been linked to the development of a form of knowledge called “noetic,” based on explicit representations endowed with an object structure and identity whose properties can be abstracted from the context in which they are embedded (Fabbro et al., 2015). Noetic awareness enables an organism to perceive objects and events and the relationships between objects and events and process them cognitively when these objects and events are not present. This information of the noetic type overlays a primary expression of mental activity defined as “anoetic,” which is essentially based on pre-representational emotional states and essentially lacks a distinction between the self and the external environment (Solms and Panksepp, 2012). The stage of noetic representation reveals itself as a development of focused attention, which enables the differentiation and categorization of specific features of the environment and the appearance of a form of explicit object awareness and semantic and conceptual memory (Tulving, 1985). In retrospect, this is the form of consciousness Edelman defines as “primary consciousness” and that is associated with the activity of the reentrant thalamocortical circuits (Edelman, 1992) present in mammals, birds, reptiles, and children from the age of 3 months (Edelman et al., 2005).

As Hobson (2001) argued, the capacities of focused attention that allow noetic cognition to appear to be linked to monoaminergic neurotransmission (serotonin and noradrenaline) and the activity of mesencephalic nuclei such as the locus coeruleus and raphe dorsalis. On the contrary, the type of unfocused exploratory activation seems to be more related to cholinergic neurotransmission promoted by the activity of pontine nuclei such as the pedunculopontine nucleus and the dorsolateral tegmental nucleus. Cholinergic and monoaminergic transmission would therefore preside over two different forms of psycho-behavioral activity. The first, older in neuro-evolutionary terms, is based on a fluid and unfocused exploration of the environment, and therefore lacks explicit references to objects and contexts. On the other hand, the second is linked to the evolution of the cerebral cortex and the possibility of generating objective representations of the surrounding environment, i.e., closed structures with their internal shape and organization recognized as independent of themselves (see Appendix A). This type of “mind wandering” is fed by images, representations, and thoughts that have a more open and flexible form and better correspond to the affective states of the self (Alcaro and Panksepp, 2011; Christoff et al., 2016; Alcaro and Carta, 2019).

As previously suggested in other contributes (Alcaro and Panksepp, 2011; Alcaro and Carta, 2019), mind wandering is sustained by the SEEKING drive when such disposition is not constrained by focused, attentive mechanisms which restrict explorations around specific boundaries of perceptual or mental objects. This phenomenon is very clearly observed in the REM sleep phase, which is associated with the ability to dream (Hobson, 2009; Hobson and Friston, 2012) and is characterized by a remarkable activation of the ML DA system (Solms, 2000; Perogamvros and Schwartz, 2012). Functional neuroimaging studies have clearly shown that unfocused mental exploration is associated with resting-state brain activity. Intrinsic and spontaneous activity of this kind appears in some cortical and medial subcortical regions when the organism is not actively interacting with the environment (Northoff et al., 2006; Alcaro and Panksepp, 2011; Raichle, 2015; Alcaro and Carta, 2019). The ability of the mind to detach itself from the immediate requirements of the physical environment to support endogenous activity presides over the evolution of the so-called “reflexive function,” or “mentalization,” on which all the most advanced processes of communication and social interaction depend. Several empirical facts suggest that the SEEKING/Exploration system influences this type of mental activity. Indeed, the neural substrates of the SEEKING/Exploration system are implicated in all forms of unfocused mental exploration, such as creative-divergent thinking, cognitive flexibility, insights, and associative thinking (Flaherty, 2005; Chermahini and Hommel, 2010; Takeuchi et al., 2010; Zabelina et al., 2016; Boot et al., 2017). Furthermore, dysfunction of the SEEKING emotional system can lead to thinking that is characterized by a loss of motivation and the dominance of depressive rumination (Northoff et al., 2011), while lesions of the same system may lead to the disappearance of dream activity and confusion in mental life (Kaplan-Solms and Solms, 2000).

From the arguments presented above, we can hypothesize that the predisposition to addictive behavior is associated with a disturbed ability of the SEEKING/Exploration system to maintain spontaneous and unfocused activation. Loss of autonomy of SEEKING/Exploration system shows up as an impoverished intrinsic and spontaneous functioning of the mind-brain. In support of this hypothesis, recent studies have shown that patients with addictive disorders exhibit a characteristic alteration in resting-state brain activity and the intrinsic functional connectivity of medial cortical structures (Ma et al., 2011, 2015; Li et al., 2016).

With the functional deficit of the ML DA-SEEKING, a restricted cortico-centric pattern of brain activity, inseparably linked to the representation of objects or the implementation of procedural habits, strictly controls the SEEKING system and the intrinsic dynamism of the mind-brain. Coincidentally, the analysis of the dreams of patients with drug addiction reveals a gradual impoverishment of the content of dream fantasies which lose their complexity and end up focusing almost exclusively on the simple hallucinatory satisfaction of drug cravings (Colace, 2014). In contrast, the practice of meditation (mindfulness), and other forms of focused attention, have been shown to have beneficial effects in treating addictions, especially in reducing craving (Chiesa and Serretti, 2014; Ashe et al., 2015; Tapper, 2018).

## Addiction and the Socio-Cultural Context

Neuroscientific research considerably increased our knowledge of the neurobiological substrates of addiction. However, this knowledge has yet to be translated into significant advances in clinical outcomes. As recently underlined by Healing and collaborators, “*one possible reason for the disconnection between addiction neuroscience research and clinical advances (is) the relatively limited extent to which social factors have been integrated into neurobiological addiction research*” (Heilig et al., 2016, p. 592). Indeed, although human and animal studies converge in indicating that social factors play an important role in the development and maintenance of compulsive habits (Pelloux et al., 2019), new bio-psycho-social models of addiction are needed to define efficacious therapeutic strategies.

Social epidemiology on drug abuse has evidenced a strong link between addiction and poor social integration (Berkman and Kawachi, 2000; Berkman et al., 2014). Such evidence has been confirmed by animal models, since single-housed animals are considerably more predisposed to develop and maintain addiction compared to animals raised with conspecifics in an enriched environment (see Pelloux et al., 2019 for a review). Interestingly, social deprivation has been also associated with altered ML DA transmission, as well as with higher impulsive and risk-taking behaviors which usually predispose to develop addiction (Martinez et al., 2010; Li et al., 2017; Walker et al., 2019).

It may then be speculated that socio-relational deprivation (isolation, exclusion, marginalization) may impact the functional organization of the ML DA-SEEKING system, that become less open to social stimuli and more dependent on non-social sources of stimulation. Indeed, as reported by Panksepp and his collaborators, positive socio-emotional systems (PLAY and CARE systems) are evolutive branching of the SEEKING drive oriented toward characteristic kinds of social interactions (Panksepp and Biven, 2012; Tanaka et al., 2018). In accordance with such hypothesis, new therapeutic strategies directed to social integration and relational rehabilitation should be integrated with current pharmacotherapies and de-conditioning techniques to ameliorate clinical outcomes (Heilig et al., 2016).

The recognition of the relevance of socio-relational contexts suggests that the enormous impact of addiction on contemporary societies may be partially related to the lifestyle and cultural ground of advanced capitalist societies. Indeed, although drugs and alcohol had been used since ancient ages (Crocq, 2007), some authors claim that widespread drug abuse has occurred mainly in recent centuries (Westermeyer, 1999; Singer, 2012). Moreover, the problem seems still more pervasive today when considering new behavioral addictions, i.e., pathological gambling, compulsive buying, Internet addiction, etc (Grant et al., 2010).

According to Alexander (2000, 2001, 2012): “*The currently dominant paradigm assumes that addiction is either an individual disease or an individual moral breach. But this individually oriented paradigm has failed. Instead, addiction needs to be understood socially, as a way that large numbers of people adapt to the breakdown of psychologically sustaining culture under the global influence of free-market society.*” Indeed, Alexander claims that “*addiction is endemic in western free-market society [*…*] because free markets inevitably dislocate people from traditional sources of psychological, social, and spiritual support, and because “dislocation,” in this broad sense of the term, is the precursor of addiction.”*

Although Alexander’s position is not yet supported by strong evidence-based quantitative data, we think that his speculation may encourage the emergence of new research in the socio-affective neuroscience of addiction. Indeed, the lifestyle of capitalist societies may promote compulsive behaviors by forcing individuals to pursuit individualistic and competitive goals and focus on achieving immediate rewards, while depriving them of psycho-environmental contexts adequate to open the SEEKING/Explorative disposition to the socio-relational field.

A notable example of the lack of open social exploration in a highly technologized society is the widespread deprivation of children’s spontaneous play, the unstructured and unregulated activity in which children engage when left to express themselves freely, without the strict control of adults (Frost, 2009, 2012; Chudacoff, 2012). Spontaneous play among juveniles is an essential feature of the behavioral development of many different species, including humans, non-human primates, other mammals, and birds (Burghardt, 2005). It is, above all, a source of joy, i.e., an emotional state crucial for psychological well-being and growth.

Many studies have also shown that spontaneous play is at the forefront of the development of essential cognitive, emotional, and social functions and that its expression influences the activity of the frontal and prefrontal cortex, which is responsible for the balance of personality (Panksepp and Biven, 2012). Other studies have directly assessed the effects of play deprivation in juvenile animal models, showing that it disrupts neural circuits regulating aggression and stress responsivity (Kyle et al., 2019).

Unfortunately, today’s society tends to limit children’s activities to places and contexts (homes, schools, gyms, etc.) that restrict or completely prevent their free expression (Lorenzoni, 2014). To manage the inevitable build-up of frustration resulting from the impediment of this natural, energetic inclination for SEEKING/Exploration and unstructured play, today’s society offers quick and reassuring remedies: televisions, video games, social networks, candies, etc. In this way, the “fetish” penetrates modern education as a substitute for the infinite, limitless potential of SEEKING/Exploration inhibited and imprisoned in the world of objects.

Some of the reasons for this deprivation of natural, spontaneous play can also be traced to how contemporary society constructs its urban landscapes as spaces that are less and less suitable for children to express themselves freely. At the same time, such deprivation is probably linked to the now deep-rooted stress and anxiety that pervades the minds of adults when children express their natural vitality in a playful way. Such concerns, unshaped and unexpressed, may be related to a fundamental loss. The loss of the meaning of one’s existence cannot arise perhaps precisely because the disposition for SEEKING/Exploration is trapped within the prison of object-related goals and habits.

## Conclusion

This article describes and interprets the phenomenon of pathological addiction from a neuro-psycho-evolutionary perspective as an expression of vulnerability related to biological and social factors. More specifically, the predisposition to develop addiction is interpreted as a loss of functional autonomy of the SEEKING/Exploration system and a related decline in the spontaneous unfocused mode of mental activity. Consequently, cognitive processes become increasingly fixed and associated with automatic patterns of neuronal activity, which are difficult to extinguish or modify. Although most current treatment methods prefer interventions directed toward the individual using drugs or psychotherapy, the growth of the phenomena of pathological addictions inevitably forces a critical reflection on the socio-cultural conditions in which they develop.

## Author Contributions

AA had the original idea and coordinated the work. AB revised the manuscript and improved the language style. DC gave his personal contribute with special emphasis to animal research studies. All authors contributed to the article and approved the submitted version.

## Conflict of Interest

The authors declare that the research was conducted in the absence of any commercial or financial relationships that could be construed as a potential conflict of interest.

## Publisher’s Note

All claims expressed in this article are solely those of the authors and do not necessarily represent those of their affiliated organizations, or those of the publisher, the editors and the reviewers. Any product that may be evaluated in this article, or claim that may be made by its manufacturer, is not guaranteed or endorsed by the publisher.

## Appendix

### Appendix A: An Excursion Into a Neuro-Psychoanalytic Model

Our affective, neuro-ethological view of the SEEKING drive and its involvement in pathological addiction share many points in common with some recent thinking from neuro-psychoanalysis. Indeed, to a degree, the SEEKING disposition can be linked to the instinctive emotional force involved in mental energy (“libido”) and its projection toward “external objects” (Kaplan-Solms and Solms, 2000; Alcaro and Panksepp, 2011).

According to the well-known psycho-hydraulic model of drives developed by Freud, psychic energy (libido) is fueled by physical needs and leads naturally to the SEARCH for objects that can satisfy these needs and thus eliminate the conditions of homeostatic imbalance (Freud, 1915; Denton, 2006). This model does not necessarily imply that the individual is aware of the goal object being sought. Nor that this object is unconsciously represented in the mind before it appears in the perceptual field. However, suppose the individual is exploring the environment. In that case, it is possible that reaching a stimulus, or a complex of stimuli, during the act of consumption may influence the activity and limit the field of SEARCH to the object in question. Some years after Freud, Carl G. Jung also hypothesized that there is a form of emotionally motivating energy that is not focused on achieving a predetermined goal. This belief became one of the main reasons for his disagreement with Sigmund Freud. The latter believed that the libido was exclusively oriented toward the satisfaction of biological (especially sexual) drives. The former thought that the libido is an undifferentiated drive that is temporarily bound to specific purposes or objects. By its very nature, it moves freely from object to object and is constantly changing (Jung, 1956).

From the origins of psychoanalysis, Freud (1900) distinguished two forms of mental activity: focused and unfocused, defining them, respectively, as the primary and secondary thinking processes. The secondary process dominates our thinking during the waking state. The primary, ontogenetically archaic form reveals itself in dreaming and several other altered states of consciousness, such as psychedelic and religious experiences, psychosis, prodromes of epileptic crisis, meditation, creative, free-association, and magical thinking, among others (Carhart-Harris and Friston, 2010; Carhart-Harris et al., 2014).

From this novel neuro-psychoanalytic perspective, the phenomenon of addiction appears as the consequence of a robust restriction of the spontaneous expression of the unfocused primary process of thinking exerted by a repetitive and ultra-specific set of memories and procedures.

According to the early psychoanalysts, the restriction of the motivational landscape to a particular set of habits and memories was conceived of as “a compulsion to repeat” (Freud, 1920/1955), whose expression displaces the natural inclination to maintain one’s physical and psychological well-being. According to Jung (1959), addiction is governed by the action of complexes of memories and procedural habits (cognitive and motor). Such complexes can operate autonomously, i.e., independently of the ordinary will and intentions, that would normally orient behavior if such complexes were not active.

From this viewpoint, the addict is essentially conditioned, dominated by a set of internalized representations and habits that orient and channel motivational drives in increasingly specific directions. Just as Konrad Lorenz’s duck cannot avoid following the object of its imprinting, so the addict cannot avoid continually pursuing the object of addiction, thus losing the very essence of subjective existence, the capacity for self-determination. It follows, therefore, that the crucial question is to understand how such complexes of “dependent memories” are formed and how they act to influence behavior.

Based on Bowlby’s (1969/1982) studies, we can identify two primary sources of environmental deprivation that may predispose an individual to develop an addiction. First is the lack of sufficiently strong and stable socio-affective ties, capable of providing a secure base of attachment or a system of belonging. Second is the lack of psycho-environmental conditions that can foster separation, emancipation, and individual autonomy. These two deficits, through seemingly antithetical, are probably complementary aspects of the same process. Attachment studies indicate that exploratory behavior is suppressed in the absence of a sufficiently good, stable, and consistent experience with a caregiver that favors an internalized secure base (Bowlby, 1969/1982). Indeed, the lack of adequate psycho-environmental contexts may be associated with a growing difficulty in tolerating absence, emptiness, and lack as constitutive elements of human life essential for the expression of the capacity to reflect on experience (Bion, 1962; Fonagy and Target, 1997). According to Daniel Stern, it is not what happened in the past but rather how it is remembered and reconstructed in the present (Stern, 1995) that matters in the attachment relationship. Therefore, it looks plausible to imagine that the support and enhancement of a healthy exploratory function may have repercussions on representing a more coherent, stable, and healthy attachment relationship.

To use a mythological metaphor, we can consider the figure of Ulysses as the personification of the relationship between the exploratory drive and the bond of attachment. Ulysses’ journey constantly feeds on his firm intention to return to Ithaca to his wife, Penelope. At the same time, his bond with his wife and native land is maintained precisely by his continuous wandering and, therefore, the absence of the person and land he loves. Similarly, psychoanalytic literature from Melanie Klein onward suggests that the relationship with the “loved object” can be said to be complete and in a certain sense real only when one can tolerate and represent its absence, its shortcomings, and its defects. When one cannot accept the experience of being separated, one cannot really be in a relationship because a symbiotic undifferentiated state dominates the panorama of experience.
